# Are we ready to consider lung recruitability during mechanical ventilation of neonates?

**DOI:** 10.1038/s41390-025-04332-2

**Published:** 2025-08-13

**Authors:** Tobias Becher, Martijn Miedema, David G. Tingay

**Affiliations:** 1 Department of Anesthesiology and Intensive Care Medicine, University Medical Center Schleswig-Holstein, Kiel, Germany; 2 Department of Neonatology, Amsterdam UMC, University of Amsterdam, Amsterdam, The Netherlands; 3 Amsterdam Reproductive and Development Research Institute, Amsterdam, The Netherlands; 4 Neonatal Research, Murdoch Children’s Research Institute, Parkville, VIC Australia; 5 Department of Paediatrics, University of Melbourne, Melbourne, VIC Australia; 6 Department of Critical Care, University of Melbourne, Melbourne, VIC Australia

Achieving lung protection during mechanical ventilation of preterm neonates remains elusive, with rates of respiratory complications stubbornly high. The causes of ventilator-induced lung injury, such as volutrauma, atelectasis and oxygen toxicity, are well understood. Despite this, identifying and then rectifying each injurious state at the bedside is not easy. An open lung strategy has been proposed as one type of lung recruitment manoeuvre to address atelectasis, optimise lung volume, and reverse intra-pulmonary shunt.^1^ In neonates, open lung strategies using oxygenation to guide lung volume response during a series of stepwise pressure changes are well described during high-frequency oscillatory ventilation (HFOV). Importantly, they have been shown to uniformly recruit the lung and allow the clinician to identify the optimal applied pressure for oxygenation and lung mechanics.^2–4^ Oxygenation as a proxy of lung volume is not without its limitations.^5,6^ Specifically, oxygenation lacks the subtlety to identify overdistension before it is severe. More broadly, oxygenation alone may not allow the clinician to understand whether a diseased lung is suitable for recruitment (‘recruitability’).

There has been considerable interest in alternative methods of defining the lung’s response during recruitment manoeuvres, with transcutaneous carbon dioxide (TcCO_2_), lung ultrasound and electrical impedance tomography (EIT) all described.^3,6,7^ Preliminary or preclinical studies in ‘pure’ settings have suggested that EIT, with its ability to define regional ventilation and aeration states in real-time, holds appeal.^3,8^ Thus, it is with interest that Werther and colleagues report their clinical study of a new EIT parameter to define lung recruitment in this edition of *Pediatric Research*.^9^ The authors report their observations in 56 open lung strategy recruitment manoeuvres during HFOV in 47 preterm infants born less than 28 completed weeks of gestation and studied at a median (range) of 4.5 (1, 23) days after birth. In each infant, an open lung strategy was performed using oxygen to guide continuous distending pressure (CDP) steps. The median CDP prior to the open lung manoeuvre was 11 cmH_2_O, with an opening (maximum) CDP of 20 cmH_2_O and optimal CDP of 10 cmH_2_O, values consistent with previous reports.^4^ Concomitant TcCO_2_ and EIT measures were made at each pressure step. Thereafter, a novel parameter called the ‘median oscillations in aerated lung regions’ (MOR) was calculated post hoc from the EIT data. The authors found that MOR and oxygenation did not always align with regard to lung recruitment response. When there was discordance, the authors found that TcCO_2_ changes more closely matched MOR, suggesting that MOR was able to identify the difference between overdistension and recruitment.

**Fig. 1:**
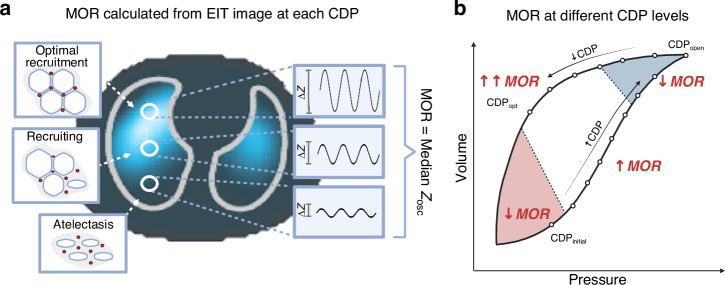
Median oscillations in the aerated regions (MOR) as a measure of lung recruitment. MOR is a calculated parameter from the EIT image at each continuous distending pressure (CDP) during an open lung strategy. The magnitude of tidal oscillations (Δ*Z*) in each air-containing pixel is determined and the median for all aerated regions calculated (Δ*Z*_osc_) and normalised (**a**). The MOR at different CDP (circles) will be dependent on the lung volume state and thus recruitability of the lung between CDP and across an entire lung recruitment manoeuvre (**b**). MOR will be impeded when the lung is atelectatic (red) or overdistended (blue), and best when homogenously recruited (near CDP_opt_).

The MOR is based on the premise that different lung states have different compliance and will generate different tidal volumes when exposed to the same pressure change. For example, an atelectatic lung region will have minimal tidal oscillations, whilst an optimally recruited region has high tidal oscillations at the same pressure amplitude (Δ*P*; Fig. 1). As EIT can measure tidal oscillations in many regions at once, the pattern of lung states can be assessed at each CDP.^10^ This can be simplified to a single value (Δ*Z*_osc_) by calculating the median of all the tidal oscillations. Δ*Z*_osc_ is then compared between the lung at standardised CDP levels, in this case, the initial CDP (when the lung is the most atelectatic) and opening CDP (the highest volume, but is also most likely to be overdistended). By normalising to body weight and using a series of standardisation rules, the authors then limit the comparisons to lung regions that contained air. The authors contend that not only does the MOR overcome known limitations of EIT (patient movement, belt displacement and signal noise), but this approach penalises overdistended regions and those that remain collapsed at all CDPs.

Randomised controlled trials in preterm infants comparing HFOV to conventional ventilation have shown inconsistent results, with no overall differences in mortality but a small trend toward lower BPD incidence with HFOV.^1^ Importantly, though, the benefit was greatest when HFOV was applied with an effective ‘high-lung volume strategy’, and least when HFOV was not (especially if lung protection was optimised during conventional ventilation).^1^ A high-lung volume strategy prioritises achieving recruitment during HFOV. But it remains uncertain whether a high-volume strategy, designed to recruit all atelectatic regions, is truly necessary, due to the required high opening CDP and risk of overdistension in healthier lung areas. A more moderate open lung volume approach could be even more effective in minimising atelectrauma without exposing the lungs to volutrauma.^8^ Studies in adults and neonatal animal models have also shown that lung regions can be either recruitable or non-recruitable.^11,12^ This important discrimination is often overlooked at the bedside. Werther and colleagues found that oxygenation suggested that 76.8% of lungs were recruitable, whilst MOR defined only 41.1% as recruitable. They further observed that the lungs defined as non-recruitable by MOR were more likely to have a TcCO_2_ and *V*_T_ response suggestive of overdistension during the open lung strategy, supporting MOR as a potentially more precise tool.

In adults, the concept of recruitable and non-recruitable lungs is well accepted, especially in the widely heterogeneous acute respiratory distress syndrome (ARDS). It has been speculated that this heterogeneity accounted for the conflicting results of HFOV trials in ARDS, causing inappropriate lung volumes from standardised CDP protocols for many patients.^13^ If so, the same has likely impacted the inconsistent trial results in preterm neonates. Inappropriate lung volumes contribute to excessive mechanical power applied at the alveolar level compared to conventional ventilation.^14^ Mechanical power describes the relationship of energy transfer between the ventilator and diseased lung, and can be considered a unifying parameter for the different injurious events within a lung. Therefore, inappropriate oscillatory settings may counteract the lung protective potential of low tidal volumes during HFOV. There is no accepted method of calculating mechanical power during HFOV, especially via an uncuffed endotracheal tube during spontaneous breathing. Hence, assessment of MOR using the method described by Werther and co-workers may serve as a tool for calculating regional power at an alveolar level during HFOV. We hope the authors will progress their work to consider the ability to use a simple EIT parameter to not just individualise adjustment of HFOV settings via considering the magnitude of regional recruitment and overdistension, but also the mechanical power implications.

The use of EIT to identify different responses to lung recruitment manoeuvres is not new. EIT has the advantage of being radiation-free, simple to use and providing continuous measures of ventilation, aeration and compliance in real-time. As it generates regional data, EIT can specify lung regions that become overdistended, are well-recruited, partially recruit (recruit during inflation but de-recruit again during tidal expiration) and those that remain collapsed.^10^ EIT systems are now commercially available for use in neonates, although use is not widespread. None yet offers MOR. This is a barrier to the generalisation of this study, especially as current EIT systems already offer simple measures of ventilation and lung volume states, such as the centre of ventilation.^10^ More importantly, most neonatal evidence comes from observational studies and interventional trials demonstrating benefit and safety over oxygenation are lacking.

The authors’ primary conclusion is that MOR may allow clinicians to distinguish between CDP changes that are causing lung recruitment versus those causing overdistension. This has a physiological plausibility, as MOR is essentially a proxy measure of lung compliance. We have previously advocated for clinicians to use some measure of compliance in addition to oxygenation during lung recruitment manoeuvres in neonates, and especially during open lung strategies.^1^ TcCO_2_, *V*_T_ and minute ventilation have all been proposed.^5^ Whether MOR is better than these cannot be concluded from this study. Even if MOR was available on commercial EIT systems, clinicians would need to balance the technical and ease-of-use pros and cons of each method in the overall care of the neonate. We would advocate that ideally, more than one approach is used at the commencement of an open lung strategy, and the least useful methods are abandoned as indicated.

Precision monitoring is essential for optimising HFOV settings and for ensuring the best possible clinical outcomes. EIT is currently the most promising bedside tool for achieving this. Using EIT for the assessment of MOR could prove to be an important step in this direction. In the meantime, this study serves as an important reminder that clinicians need to consider both how to recruit a diseased neonatal lung and how to best measure when that lung is not recruitable.
